# Effects of Citrus Flavanone Hesperidin Extracts or Purified Hesperidin Consumption on Risk Factors for Cardiovascular Disease: Evidence From an Updated Meta-analysis of Randomized Controlled Trials

**DOI:** 10.1016/j.cdnut.2023.102055

**Published:** 2023-12-09

**Authors:** Haohai Huang, Dan Liao, Bin He, Guanghui Zhou, Yejia Cui

**Affiliations:** 1 Clinical Translational Medical Center, The Affiliated Dongguan Songshan Lake Central Hospital, Guangdong Medical University, Dongguan, Guangdong, China; 2 Department of Clinical Pharmacy, The Affiliated Dongguan Songshan Lake Central Hospital, Guangdong Medical University, Dongguan, Guangdong, China; 3 Department of Gynaecology, The Affiliated Dongguan Songshan Lake Central Hospital, Guangdong Medical University, Dongguan, Guangdong, China; 4 Department of Rehabilitation Medicine, The Affiliated Dongguan Songshan Lake Central Hospital, Guangdong Medical University, Dongguan, Guangdong, China; 5 Department of Clinical Laboratory, The Affiliated Dongguan Songshan Lake Central Hospital, Guangdong Medical University, Dongguan, Guangdong, China

**Keywords:** blood pressure, CVD, lipid, hesperidin, glucose, meta-analysis, inflammation

## Abstract

**Background:**

Cardiovascular disease (CVD) is a serious public health problem worldwide. The role of citrus flavanone hesperidin consumption on cardiovascular disease risk factors (CVDRFs) has been examined in many clinical trials, but conflicting results have been found.

**Objectives:**

This study aimed to systematically evaluate the effects of hesperidin extracts or purified hesperidin on CVDRFs in humans with an updated meta-analysis of randomized controlled trials.

**Methods:**

According to the Preferred Reporting Items for Systematic Reviews and Meta-Analyses 2020 guidelines, we systematically screened and searched electronic databases from their establishment to March 2023. Reference lists and previous reviews were also searched. Intervention trials assessing hesperidin consumption on CVD outcomes were included for pooling. To assess the quality of the included articles, the tool of Cochrane risk-of-bias tool was applied. We synthesized the effect sizes with 95% CIs and weighted mean difference (WMD). The *I*^2^ index was used to evaluate the between-study heterogeneity. To explore the heterogeneity source, we used meta-regression and subgroup analysis. Publication bias and sensitivity analysis were also performed. We used the Grading of Recommendations Assessment, Development, and Evaluation approach to evaluate the evidence quality.

**Results:**

We included 12 trials with 589 participants. We found evident effects of hesperidin on low-density lipoprotein cholesterol (WMD: −0.22 mmol/L; 95% CI: −0.33, −0.11 mmol/L), total cholesterol (WMD: −0.20 mmol/L; 95% CI: −0.31, −0.08 mmol/L), fasting blood glucose (WMD: −0.15 mg/dL; 95% CI: −0.29, −0.02 mg/dL), quantitative insulin-sensitivity check index (WMD 0.06, 95% CI 0.01 to 0.10), intercellular adhesion molecule 1 (WMD: −13.60 ng/mL; 95% CI: −23.72, −3.48 ng/mL), vascular cell adhesion molecule 1 (WMD: −15.60 ng/mL; 95% CI: −30.13, −1.06 ng/mL), and C-reactive protein (WMD: −0.56 mg/L; 95% CI: −1.11, −0.01 mg/L), whereas no effects were found for other CVDRFs.

**Conclusions:**

Our current findings demonstrate that hesperidin might be advantageous in improving numerous CVDRFs in humans, such as blood lipid concentrations, blood glucose control, and management of inflammatory indicators.

## Introduction

Cardiovascular disease (CVD) is a serious public health problem and is significantly connected to most disabilities, morbidities, and mortalities worldwide [1,2]. With the global aging of populations, the prevalence of CVD is increasing rapidly. Several modifiable cardiovascular disease risk factors (CVDRFs), such as inappropriate diet, inflammation, type 2 diabetes, obesity, hypertension, insulin resistance, and dyslipidemia, are related to CVD development and associated complications [[3], [4], [5]]. Previous studies have shown that controlling these CVDRFs can decrease or prevent the CVD development [6,7]. Owing to its health and financial burden on society, primary and secondary CVD prevention and delaying the occurrence and progression of CVD-related problems are crucial for public health. According to the literature from randomized controlled trials (RCTs) and epidemiology, natural dietary products and nutraceutical therapies significantly affect CVD prevention and management for many reasons, such as poor adherence to medications, drug contraindications, various side effects, or individual preference for natural or alternative therapies [8,9].

In European adults, the mean flavonoid intake is 428 mg/d [10]. Flavanones, such as hesperidin, is a bioactive plant compound that is mainly found in citrus fruits, such as lemons, clementine, grapefruit, and mandarins and their juices [11]. Hesperidin, which accounts for ∼90% of the flavanone glycosides in orange juice, is mainly concentrated in the solid parts and membranes that separate the pulp segments of citrus fruits [12]. In single-strength juice, hesperidin content ranges from 555 to 761 mg/L, whereas in concentrated juice, it ranges from 470 to 614 mg/L [13]. Supplementation with hesperidin has various benefits, such as anti-inflammatory, anticancer, and antioxidant characteristics [[14], [15], [16]]. In addition, many animal and human studies have explored hesperidin’s cardiovascular preventive benefits [[17], [18], [19]]. RCTs have also investigated the effectiveness of hesperidin consumption on various CVDRFs [[20], [21], [22], [23]]. However, very small sample sizes, different supplementation dosages, and diverse underlying health states hampered the outcomes and made them inconsistent. Two previous systematic review and meta-analyses summarized the evidence on the effects of hesperidin on CVDRFs [24,25]. The study conducted by Mohammadi et al. [24], including 10 RCTs showed that the hesperidin supplementation might not improve lipid profile and blood pressure. However, the effects of hesperidin consumption on markers related to body composition, glycemic indices, and inflammation were not evaluated in this study. Another meta-analysis conducted by Pla-Pagà et al. [25] revealed that hesperidin consumption reduces glucose concentrations and various lipid profile parameters in animal studies. However, a definitive conclusion cannot be drawn in the human clinical trials. Of note, 7 publications included in the previous meta-analysis analyzed consumption of orange juice [[26], [27], [28], [29], [30], [31], [32]], not the purified hesperidin or standardized hesperidin–enriched extracts. The compositions of the orange juice contained hesperidin, naringin, vitamin C, folate, total sugar, didymin, and citric acid. The beneficial effect of hesperidin supplementation on CVDRFs could be attributed to other nutrients consumption. Thus, it may be improper to include these studies for pooling. Overall, the previous meta-analyses might threaten the authenticity of their findings. To provide the latest and most convincing evidence, we performed a systematic review and meta-analysis of human RCTs that evaluated the consumption of citrus flavanone hesperidin extract or purified hesperidin on cardiometabolic health outcomes.

## Methods

We conducted the design, implementation, and reporting according to the PRISMA 2020 guidelines [33].

### Strategy for literature search

In this study, the keyword-based search was performed by 2 authors (HH and DL) in Cochrane Central Register of Controlled Trials, MEDLINE/PubMed, Google Scholar, Scopus, EMBASE, and Web of Science, from their establishment to March 2023, without using language restrictions, to find all eligible RCTs regarding the clinical effectiveness of hesperidin on CVDRFs in adults. The complete search strategy, including Medical Subjects Headings and free-text terms, are provided in Supplemental Table 1. Related reviews and references cited by the included articles were also screened to find more relevant articles.

### Study inclusion and exclusion criteria

After comprehensive reading of the titles, abstracts, and full texts, the following inclusion criteria were used to select eligible studies:

• Participants: adults (age >18 y);
• Intervention: subjects needed to have specifically ingested purified hesperidin or standardized hesperidin–enriched extracts; the hesperidin and other combined intervention trials were selected when these combined interventions were used as the control group, with an intervention duration of ≥2 wk;
• Comparators: no hesperidin consumption or placebo capsule (cellulose and starch) interventions or consumed substitutions containing no hesperidin;
• Outcomes: relevant cardiovascular health primary outcomes included markers related to lipid profile, blood pressure, body composition, glycemic indices, and inflammation;
• Study design: original RCTs with a parallel or crossover design.

Excluded from the analysis were duplicate data, nonrandomized study designs, animal studies, studies using an active comparator (such as grape or apple juice) in the control group, studies lacking a control group, and reviews. Furthermore, studies with ambiguous information and those lacking response from the corresponding authors despite attempts to contact them were also excluded.

### Data extraction and quality assessment

Data were retrieved by 2 researchers (GZ and BH). If the same patients were mentioned in multiple publications but with variations in reported outcomes, we included all publications. However, the sample size from these publications was only considered once during calculations. Nonagreements were resolved by a third researcher (DL). We extracted the following characteristics from each included study:

• Publication data: first author’s name, year of publication, and country in which the trial was performed;
• Participant characteristics: mean age, health condition, and sex;
• Study characteristics: enrolled and completed patient number, hesperidin dosage and formulation, control treatment type, intervention duration, and details of the study design;
• Study outcomes: means and SDs of primary outcomes at baseline, posttreatment, or changes between baseline and posttreatment. Serious adverse events were also extracted. Based on the Cochrane Handbook for Systematic Reviews of Interventions [34], SDs were estimated using the SE or 95% CIs when they were not directly available.

We applied the Cochrane risk-of-bias tool for RCTs. Criteria, such as incomplete outcome data, blinding of outcome assessment, selective reporting, blinding of participants and personnel, random sequence generation, and allocation concealment, were applied to evaluate the characteristics’ quality. According to the suggestions from the Cochrane Handbook, a qualitative degree (no, yes, or unclear) was assigned for each item, and judgment of each included study was labeled with unclear, high, or low risk of bias.

### Synthesis and analysis of data

We used the DerSimonian and Laird random-effects model to calculate the 95% CIs and the weighted mean difference (WMD) for continuous outcomes. The *I*^2^ index and the Q test evaluated the heterogeneity between trials. *I*^2^ scores of ≥50% indicated moderate to high heterogeneity [35]. A conventional analysis of sensitivity was conducted by removing 1 study at a time to examine whether the overall results relied on individual studies. Then, we performed a priori analysis of subgroup to find probable causes of heterogeneity when there were sufficient numbers of studies. The publication bias probability was examined using the asymmetry of Egger weighted regression statistics, Begg rank correlation, and funnel plot [36,37]. We used a random-effects meta-regression analysis based on the restricted maximum likelihood to determine whether the duration and amount of hesperidin supplementation were related to the heterogeneity source [38]. We used the Grading of Recommendations Assessment, Development, and Evaluation (GRADE) technique to evaluate the evidence quality for each metabolic and cardiovascular biomarker. The evidence quality was classified to very low, low, moderate, and high [39]. RevMan (version 12.0) and STATA (version 5.0) were applied for all statistical analyses. Two-sided comparisons were applied in all analyses unless stated otherwise. A significant difference was set as a *P* value of <0.05.

## Results

### Main search

**FIGURE 1:**
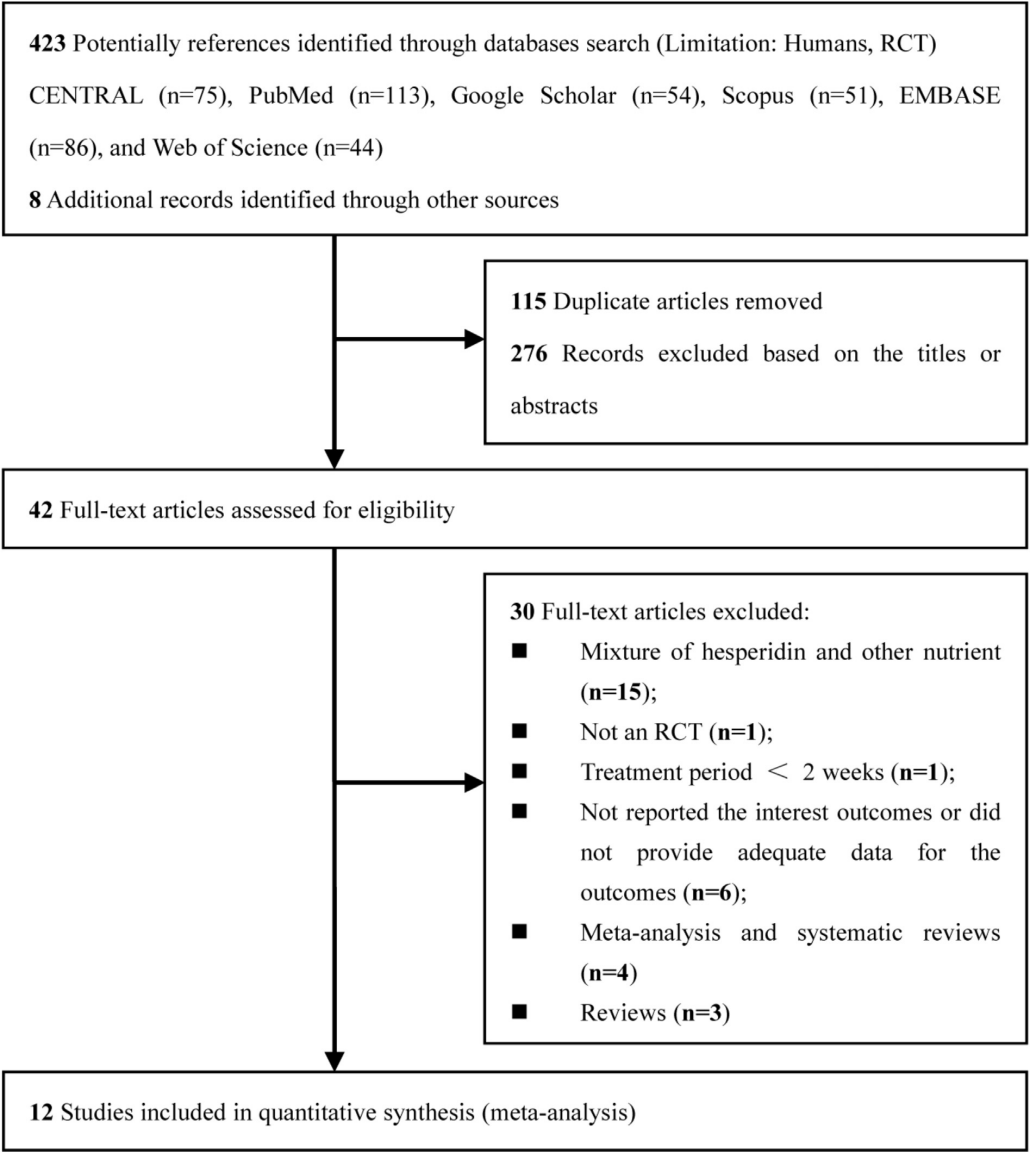
Selection of studies for inclusion. RCT, randomized controlled trial.

Based on the search strategy, 423 records were identified in Cochrane Central Register of Controlled Trials (*n* = 75), PubMed (*n* = 113), Google Scholar (*n* = 54), Scopus (*n* = 51), EMBASE (*n* = 86), and Web of Science (*n* = 44). Eight additional studies were included using reference lists from the included reviews. After duplication analysis (*n* = 115), 276 publications were excluded based on the review of titles and abstracts. We also removed reviews (*n* = 3) or meta-analyses and systematic reviews (*n* = 4), studies that did not provide adequate data for the outcomes (*n* = 6), those that did not report the primary outcomes, studies with treatment periods of <2 wk (*n* = 1), studies that were not an RCT (*n* = 1), or studies that used mixture of hesperidin and other nutrients (*n* = 15). Finally, 12 publications were included in this meta-analysis to evaluate the effects of hesperidin on cardiovascular health [[20], [21], [22], [23],[40], [41], [42], [43], [44], [45], [46], [47]]. Details of the flow diagram of study selection are shown in Figure 1.

### Subjects and study characteristics

Study characteristics and the main participant information are presented in Table 1. The number of participants in each trial ranged from 24 to 124. We included 608 participants, and 96.58% (589) completed the studies.

**TABLE 1:** Characteristics of study populations, type of interventions, and study designs in the included trials

Study	Year	Study design	Country	Sample size (enrolment/completed)	Sex (M/F)	Study participants	Mean age^1^	BMI (kg/m^2^)^1^	Intervention		Dose (mg/d)	Duration (wk)	Outcomes^1^
									Treatment group	Control group			
Salden et al. [20]	2016	R, PC, DB, P	Netherlands	68/68	29/39	Healthy overweight individuals	53.00	29.00	Hesperidin 2S	Placebo (cellulose)	450	6	VCAM-1, ICAM-1, E-selectin, SBP, DBP, TC, LDL cholesterol, HDL cholesterol, TG, FBS, insulin, QUICKI
Rizza et al. [45]	2011	R, PC, DB, C	Italy	24/24	15/9	Patients with metabolic syndrome	52.00	34.70	Hesperidin	Placebo (cellulose)	500	3	SBP, DBP, CRP, VCAM, E-selectin, BMI, WC, FBG, insulin, HOMA-IR, TC, LDL cholesterol, HDL cholesterol, TG, QUICKI
Morand et al. [44]	2011	R, PC, C	France	24/24	24/0	Healthy subjects with no evidence of chronic disease	56.00	27.4	Control drink plus hesperidin	Control drink plus placebo (starch)	292	4	SBP, DBP, FBG, insulin, TC, LDL cholesterol, HDL cholesterol, TG, CRP, ICAM-1, VCAM-1
Demonty et al. [41]	2010	R, PC, DB, P	Netherlands	124/124	65/59	Moderately hypercholesterolemic individuals	60.55	25.10	Hesperidin	Placebo (microcrystalline cellulose)	800	4	BW, BMI, TC, LDL cholesterol, HDL cholesterol, TG
Homayouni et al. [21]	2018	R, PC, DB, P	Iran	64/60	NA	Type 2 diabetes	52.85	27.75	Hesperidin	Placebo (starch)	500	6	BW, BMI, SBP, DBP, CRP
Homayouni et al. [43]	2017	R, PC, DB, P	Iran	64/60	NA	Type 2 diabetes	52.83	27.73	Hesperidin	Placebo (starch)	500	6	BW, BMI, FBG, HOMA-IR
Ohara et al. [22]	2016	R, PC, DB, P	Japan	30/29	14/15	Healthy, moderately obese subjects	49.20	26.15	Glucosyl-hesperidin	Placebo	500	12	BW, BMI, WC, HC, TC, LDL cholesterol, HDL cholesterol, TG
Cheraghpour et al. [40]	2019	R, PC, DB, P	Iran	50/49	22/27	Patients with nonalcoholic fatty liver disease	47.30	32.43	Hesperidin	Placebo	1000	12	FBS, insulin, HOMA-IR, TC, LDL cholesterol, HDL cholesterol, TG, CRP
Haidari et al. [42]	2015	R, PC, DB, P	Iran	75/75	NA	Patients with myocardial infarction	55.49	26.39	Hesperidin	Placebo (starch)	600	4	BW, BMI, WC, HC, SBP, DBP, CRP, E-selectin, TC, LDL cholesterol, HDL cholesterol, TG
Yari et al. [23]	2020	R, PC, C	Iran	49/44	23/21	Patients with metabolic syndrome	46.12	31.53	Hesperidin	No supplement	1000	12	BW, BMI, WC, TG, HDL cholesterol, FBS, insulin, HOMA-IR, SBP, DBP, QUICKI
Yari et al. [46]	2021	R, PC, DB, P	Iran	50/49	25/24	Patients with metabolic syndrome	45.19	31.31	Hesperidin	Placebo (starch)	1000	12	
Yari et al. [47]	2021	R, PC	Iran	50/43	21/22	Patients with nonalcoholic fatty liver disease	45.97	32.06	Lifestyle modification program with hesperidin supplementation	Lifestyle modification program	1000	12	BMI, WC, FBS, insulin, HOMA-IR, TC, LDL cholesterol, HDL cholesterol, TG, CRP, QUICKI

BMI, body mass index; BW, body weight; C, crossover; CRP, C-reactive protein; DB, double-blind; DBP, diastolic blood pressure; F, female; FBG, fasting blood glucose; HC, hip circumference; HDL, high-density lipoprotein; HOMA-IR, homeostatic model assessment of insulin resistance; ICAM, intercellular adhesion molecule; LDL, low-density lipoprotein; M, male; NA, not available; P, parallel; PC, placebo-controlled; R, randomized; SBP, systolic blood pressure; TC, total cholesterol; TG, triglyceride; VCAM, vascular cell adhesion molecule; WC, waist circumference.

1 Values for age and BMI are expressed as mean unless otherwise stated.

#### Publication details.

The publication date ranged from 2010 to 2020. Researchers from 5 countries performed these studies: Netherlands (*n* = 2) [20,41], Italy (*n* = 1) [45], France (*n* = 1) [44], Japan (*n* = 1) [22], and Iran (*n* = 7) [21,23,40,42,43,46,47].

#### Subject characteristics.

In each trial, the number of participants ranged from 24 to 124, and 649 individuals were finally included for data pooling. One study included only male subjects, 8 included both sexes, and 3 did not specify gender composition. The average age ranged from 45.19 to 60.55 y, and the BMI (kg/m^2^) values ranged between 25.10 and 34.70. Healthy subjects were enrolled in only 3 studies [20,22,44]. Other trials were performed among participants with various diseases, such as type 2 diabetes (*n* = 2) [21,43], nonalcoholic fatty liver disease (*n* = 2) [40,46], metabolic syndrome (*n* = 3) [23,45,47], hypercholesterolemia (*n* = 1) [41], and myocardial infarction (*n* = 1) [42].

#### Study characteristics.

The doses of hesperidin administered ranged from 292 to 1000 mg/d. Ten trials used hesperidin [21,23,[40], [41], [42], [43], [44], [45], [46], [47]], 1 used hesperidin 2S [20], and 1 used G-hesperidin [22]. Placebo control interventions included cellulose, starch, or microcrystalline cellulose. The duration of the intervention ranged from 3 to 12 wk. Four trials were designed as parallel-group studies [[44], [45], [46], [47]], and 8 used a parallel design [[20], [21], [22], [23],[40], [41], [42], [43]].

#### Outcome measurement.

In these enrolled studies, the CVDRFs included E-selectin, intercellular adhesion molecule (ICAM)-1, vascular cell adhesion molecule (VCAM)-1, C-reactive protein (CRP), waist circumference (WC), body weight (BW), hip circumference (HC), quantitative insulin-sensitivity check index (QUICKI), BMI, HOMA-IR, insulin, fasting blood glucose (FBG), diastolic blood pressure (DBP), systolic blood pressure (SBP), triglycerides (TGs), HDL cholesterol, LDL cholesterol, and total cholesterol (TC). Twelve trials investigated the effects of hesperidin on lipid profile; 8 on FBG and BMI; 7 on BP, insulin, and BW; 6 on WC and CRP; 5 on HOMA-IR and QUICKI; 3 on VCAM-1 and E-selectin; and 2 on HC and ICAM-1.

#### Trial quality evaluation.

**FIGURE 2:**
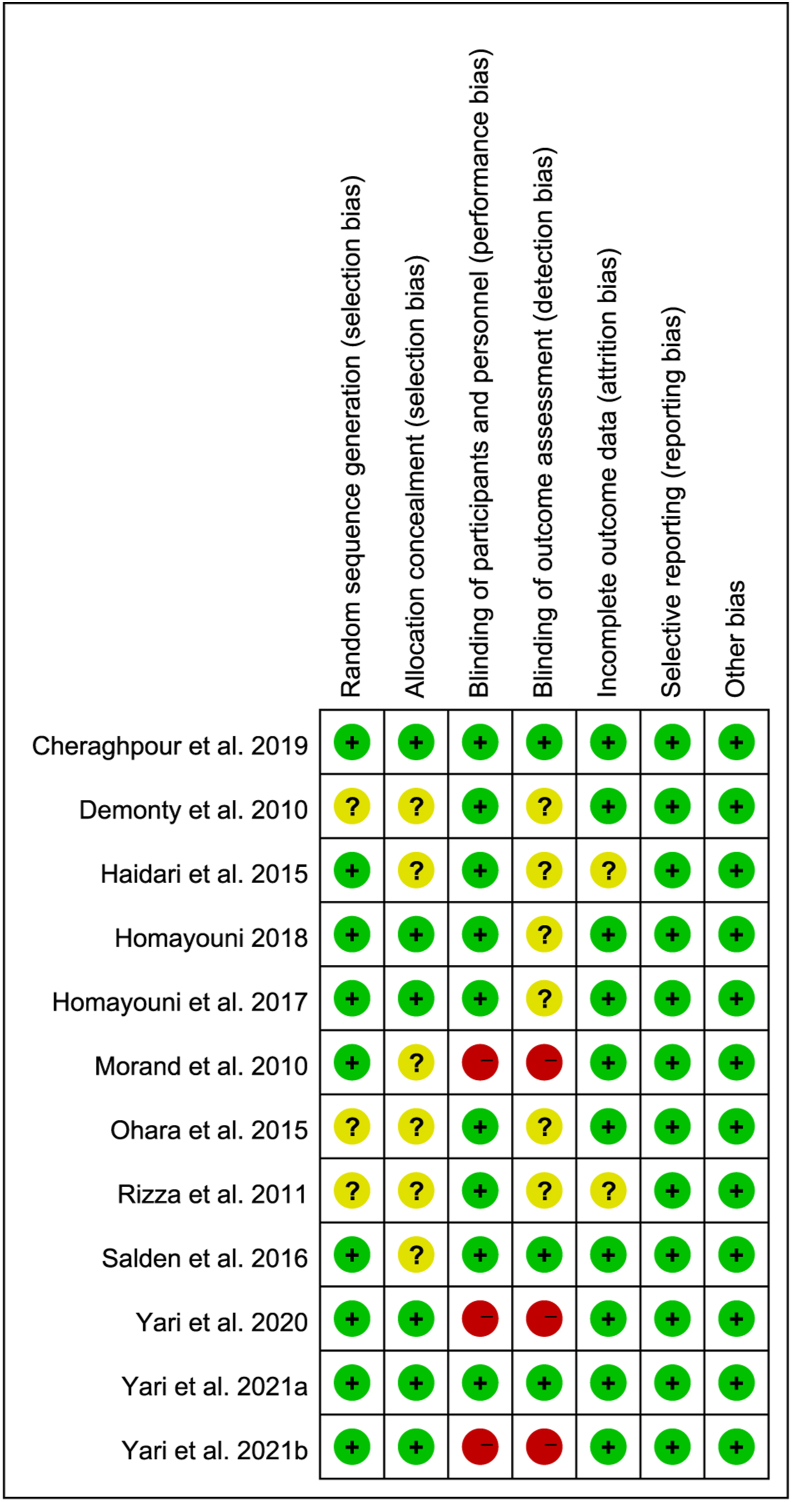
Quality assessment of the included studies. Question mark indicates an unclear or unknown risk of bias; negative sign indicates a high risk of bias; positive sign indicates a low risk of bias.

A summary of the Cochrane Collaboration tool’s risk-of-bias assessment is presented in Figure 2. A high risk of bias was found in 3 studies [23,44,47], an unknown risk was observed in 7 studies [[20], [21], [22],[41], [42], [43],^45^], and a low risk was discovered in 2 studies [40,46]. Thus, 2 studies were rated as high quality, whereas others were rated as fair and poor.

### Quantitative data synthesis from the overall meta-analysis and subgroup analysis

We included 12 studies with 589 participants on the effects of hesperidin on CVDRFs. We performed analysis of prespecified subgroup to explore the overall effects of hesperidin in CVDRFs effects, including mean age (>50 compared with <50 y), study design (crossover compared with parallel design), baseline BMI (<30 compared with ≥30), hesperidin dose (<500 compared with ≥500 mg/d), and the intervention duration (<6 compared with ≥6 wk) (Supplemental Table 2).

### Effects of hesperidin supplementation on lipid profile

**FIGURE 3:**
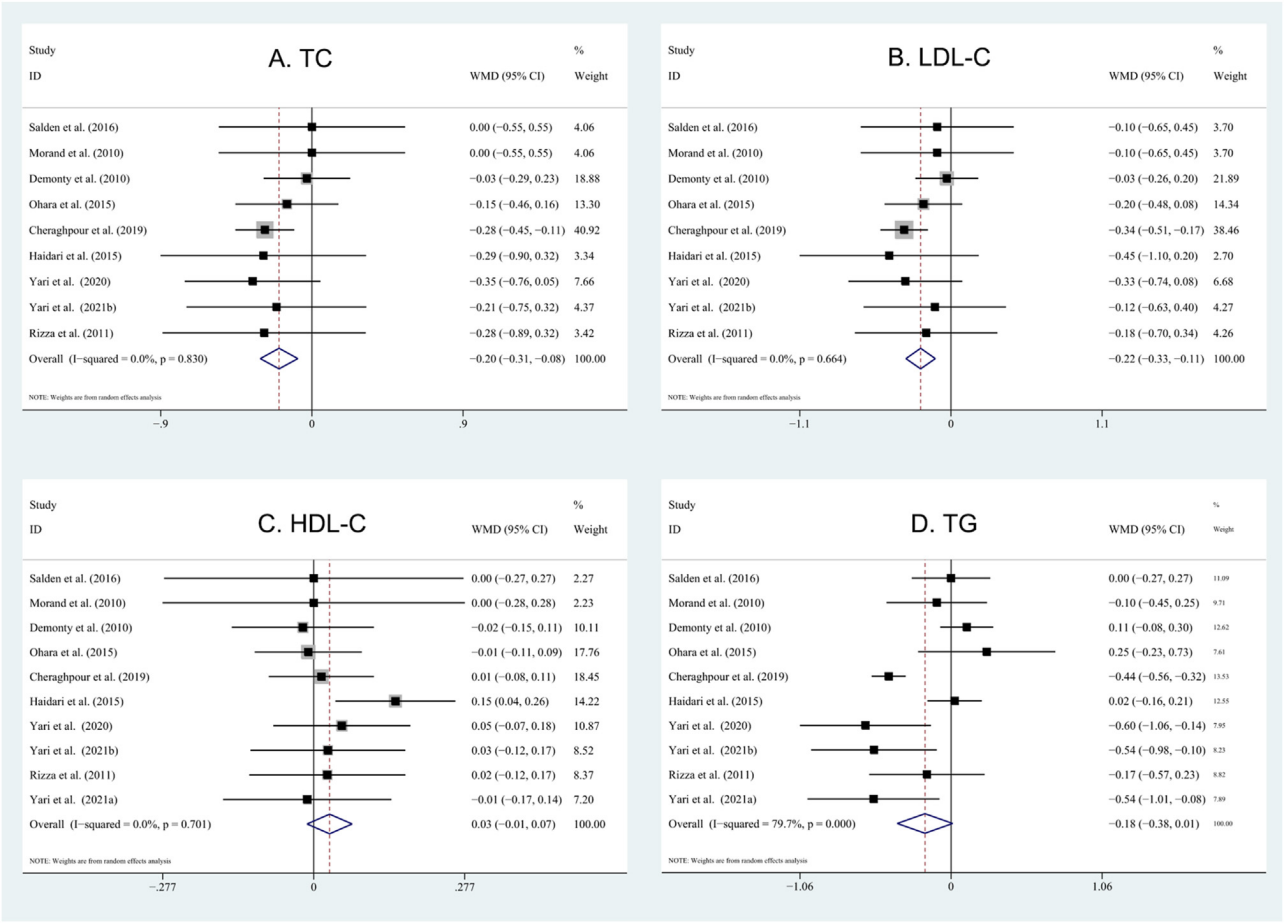
Forest plot illustrating the weighted mean difference of hesperidin on blood lipids with a random-effects model: (A) TC; (B) LDL cholesterol; (C) HDL cholesterol; (D) TG. HDL cholesterol, high-density lipoprotein cholesterol; LDL cholesterol, low-density lipoprotein cholesterol; TC, total cholesterol; TG, triglyceride.

By using a random-effects model, the pooled results suggested that hesperidin supplementation significantly reduced the concentrations of TC (WMD: −0.20 mmol/L; 95% CI: −0.31, −0.08 mmol/L; *P* = 0.0006; *I*^2^ = 0%) and LDL cholesterol (WMD: −0.22 mmol/L; 95% CI: −0.33, −0.11 mmol/L; *P* = 0.00001; *I*^2^ = 0%). However, the overall analysis showed that hesperidin did not affect HDL cholesterol (WMD: 0.03 mmol/L; 95% CI: −0.01, 0.07 mmol/L; *P* = 0.17; *I*^2^ = 0%) and TG (WMD: −0.18 mmol/L; 95% CI: −0.38, 0.01 mmol/L; *P* = 0.07; *I*^2^ = 79.7%) concentrations. A forest plot for hesperidin effects on lipid profile is shown in Figure 3. Subgroup analyses were also performed to clarify the hesperidin effects on plasma lipid levels in some prespecified subgroups. We found that hesperidin significantly reduced LDL cholesterol and TC (*P* < 0.05) concentrations in the following subgroups: hesperidin ≥500 mg/d, duration ≥6 wk, parallel design, mean BMI ≥30, and mean age of <50 y. Moreover, HC significantly decreased TG in subjects with BMI ≥30 and <50 y and trials with crossover design and duration of ≥6 wk.

### Effects of hesperidin supplementation on blood pressure

**FIGURE 4:**
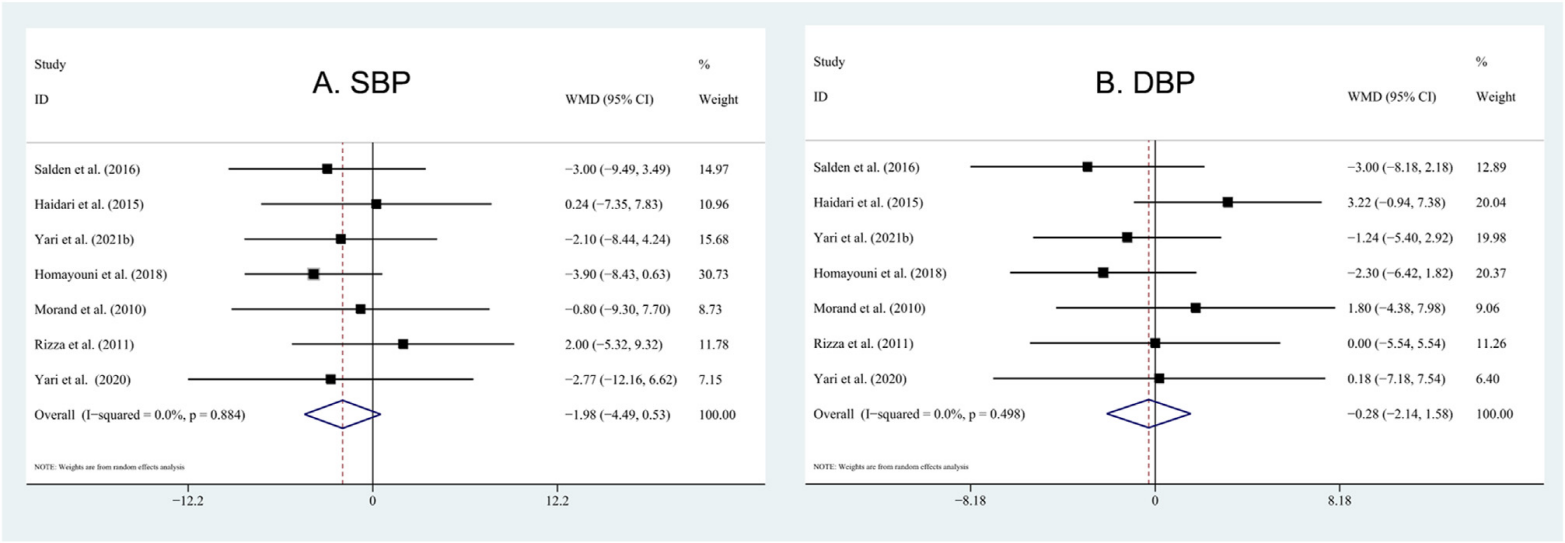
Forest plot illustrating the weighted mean difference of hesperidin on blood pressure with a random-effects model: (A) SBP; (B) DBP. DBP, diastolic blood pressure; SBP, systolic blood pressure.

Hesperidin intervention did not affect SBP (WMD: −1.98 mm Hg; 95% CI: −4.49, 0.53 mm Hg; *P* = 0.12) and DBP (WMD: −0.28 mm Hg; 95% CI: −2.14, 1.58 mm Hg; *P* = 0.77) (Figure 4). No significant heterogeneity was detected in the RCTs for SBP and DBP (*I*^2^ = 0%). Similarly, we did not find significant effects of hesperidin on SBP and DBP in the subgroup analysis based on study design, mean age, intervention duration, mean BMI, and hesperidin dose.

### Effects of hesperidin supplementation on glycemic indices

**FIGURE 5:**
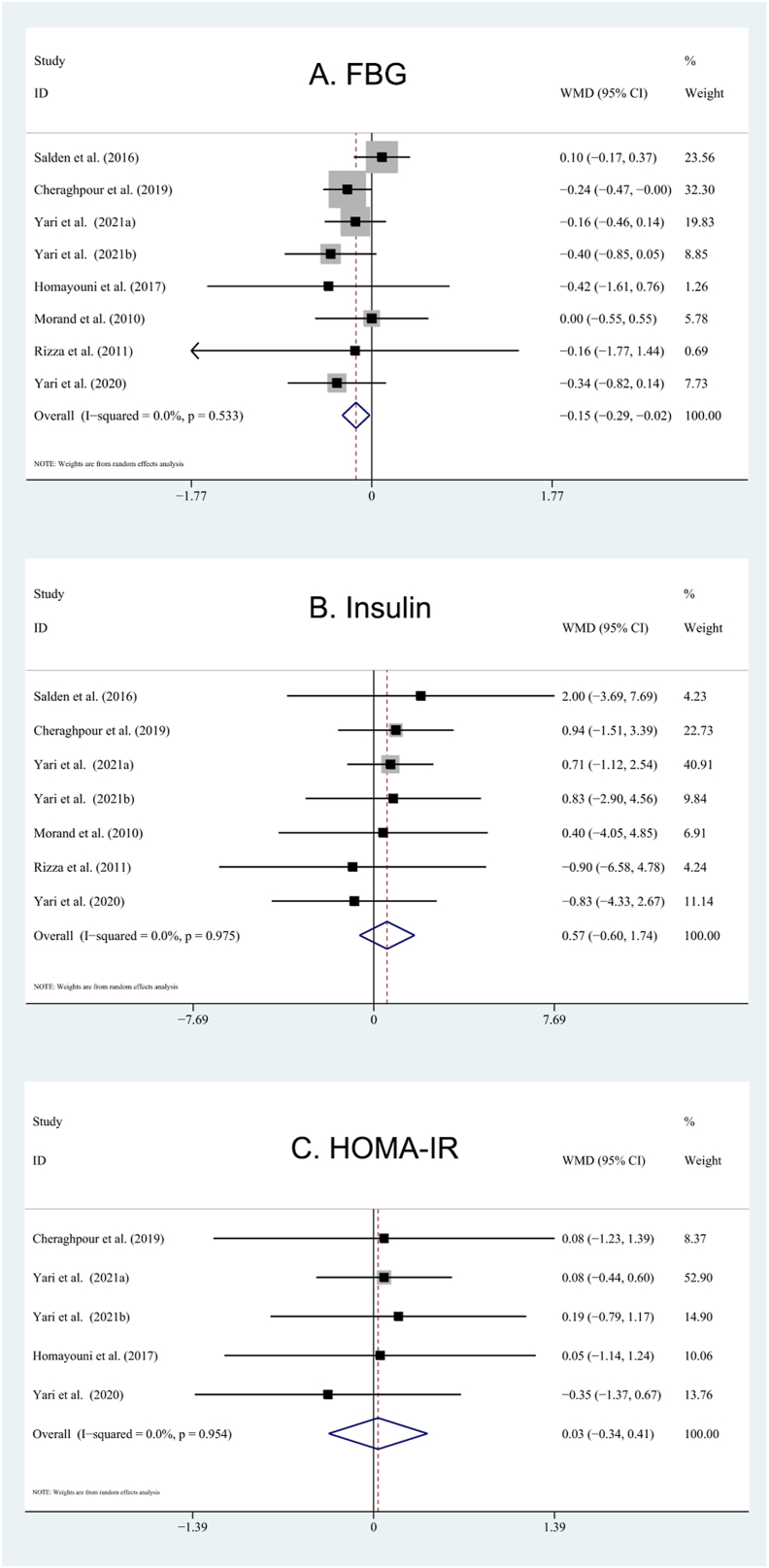
Forest plot illustrating the weighted mean difference of hesperidin on glycemic control with a random-effects model: (A) FBG; (B) insulin; (C) HOMA-IR. FBG, fasting blood glucose; HOMA-IR, homeostatic model assessment of insulin resistance.

Hesperidin had an inhibitory effect on FBG (WMD: −0.15 mg/dL; 95% CI: −0.29, −0.02 mg/dL; *P* = 0.02; *I*^2^ = 0%) and QUICKI (WMD: 0.06; 95% CI: 0.01, 0.10; *P* = 0.02; *I*^2^ = 99%). However, insulin (WMD: 0.57 μU/mL; 95% CI: −0.60, 1.74 μU/mL; *P* = 0.41; *I*^2^ = 0%) and HOMA-IR (WMD: 0.03; 95% CI: −0.34, 0.41; *P* = 0.86; *I*^2^ = 0%) did not differ compared with the control group (Figure 5). We also found a significant reduction in FBG for subjects with BMI of ≥30, subjects <50 y, dosages ≥500 mg/d, duration ≥6 wk, and crossover design. Meanwhile, we detected a significant change in QUICKI in subjects with BMI of <30 and dosages <500 mg/d.

### Effects of hesperidin supplementation on body composition and inflammatory markers

In comparison with the control group, hesperidin intake did not affect the BW (WMD: 0.08 kg; 95% CI: −0.18, −0.33 kg; *P* = 0.55; *I*^2^ = 0%), BMI (WMD: −0.05 kg/m^2^; 95% CI: −0.58, 0.47 kg/m^2^; *P* = 0.84; *I*^2^ = 0%), WC (WMD: −1.45 cm; 95% CI: −3.23, 0.33 cm; *P* = 0.11; *I*^2^ = 28%), and HC (WMD: −0.73 cm; 95% CI: −1.69, 0.22 cm; *P* = 0.13; *I*^2^ = 0%) (Table 2). Meanwhile, the subgroup analysis demonstrated that hesperidin decreased WC (*P* < 0.05) in subjects with BMI of ≥30 and trials with a crossover design.

**TABLE 2:** Effect of hesperidin consumption on body composition and inflammation markers in human subjects

Outcomes	No. of trials	No. of patients	WMD (95% CI)	*P*	*I*^2^ (%)	*P*-heterogeneity
BW	7	382	0.08 (−0.18, 0.33)	0.55	0	1.00
BMI	8	424	−0.05 (−0.58, 0.47)	0.84	0	0.95
WC	6	289	−1.45 (−3.23, 0.33)	0.11	28	0.22
HC	2	105	−0.73 (−1.69, 0.22)	0.13	0	0.79
CRP	6	324	−0.56 (−1.11, −0.01)	0.04^1^	56	0.05
VCAM-1	3	144	−15.60 (−30.13, −1.06)	0.04^1^	87	0.006
ICAM-1	2	96	−13.60 (−23.72, −3.48)	0.008^1^	62	0.10
E-selectin	3	171	−2.25 (−6.14, 1.64)	0.26	59	0.09

BMI, body mass index; BW, body weight; CRP, C-reactive protein; HC, hip circumference; ICAM, intercellular adhesion molecule; VCAM, vascular cell adhesion molecule; WC, waist circumference.

1 Indicates a significant result.

We also evaluated the effects of hesperidin ingestion on inflammation markers, such as VCAM-1, ICAM-1, E-selectin, and CRP. Hesperidin significantly reduced CRP (WMD: −0.56 mg/L; 95% CI: −1.11, −0.01 mg/L; *P* = 0.048; *I*^2^ = 56%), VCAM-1 (WMD: −15.60 ng/mL; 95% CI: −30.13, −1.06 ng/mL; *P* = 0.04; *I*^2^ = 87%), and ICAM-1 (WMD: −13.60 ng/mL; 95% CI: −23.72, −3.48 ng/mL; *P* = 0.008; *I*^2^ = 62%). However, hesperidin did not affect E-selectin (WMD: −2.25 ng/mL; 95% CI: −6.14, 1.64 ng/mL; *P* = 0.826; *I*^2^ = 59%) (Table 1). In addition, we found a significant reduction in CRP for subjects with BMI of ≥30, subjects aged <50 y, dosages ≥500 mg/d, duration ≥6 wks, and crossover design.

### Clinical safety evaluation

No serious adverse effects were found in most trials after hesperidin consumption, suggesting a good tolerance to hesperidin. Only 1 RCT recorded adverse effects during the study period. Ohara et al. [22] reported that 6 participants receiving G-hesperidin (500 mg/d) presented symptoms of a common cold (*n* = 3), pain (*n* = 2), and conjunctivitis (*n* = 1).

### Sensitivity analyses

We also conducted sensitivity analyses to verify the robustness of the results. The pooled results or overall effect size for CVDRFs remained solid and were not sensitive to any individual study. Moreover, after using the fixed-effects model, the analysis of sensitivity revealed that the combined effects of hesperidin on CVDRFs did not significantly change (Supplemental Table 3).

### Dose–response relation between doses and duration of hesperidin intake and outcomes

Because hesperidin was supplied at various doses and durations in the included trials, the correlation of changes in CVDRFs with hesperidin dosage (milligrams per day) and duration (weeks) were evaluated (Supplemental Table 4). We did not detect a between the duration of supplementation and the impact of hesperidin on CRP, WC, BMI, BW, insulin, FBG, DBP, SBP, HDL cholesterol, LDL cholesterol, and TC (*P* > 0.05). Meanwhile, the hesperidin amount consumed per day was not related to changes in CRP, WC, BMI, BW, Insulin, FBG, DBP, SBP, HDL cholesterol, TC, and LDL cholesterol (*P >* 0.05). However, the meta-regression analysis showed that the efficacy of hesperidin in lowering TG concentrations significantly increased with increasing doses (coefficient: −0.0007; 95% CI: −0.0014, −0.0008; *P* = 0.031) and duration (coefficient: −0.0563; 95% CI: −0.0841, −0.0286; *P* = 0.002). We did not perform a dose–response relation test for HOMA-IR, QUICKI, HC, ICAM-1, VCAM-1, and E-selectin owing to insufficient data (*n* ≤ 5).

### GRADE evidence profile assessment

The GRADE profiles for the clinical evidence of hesperidin consumption on blood lipids, blood pressure, glycemic indices, inflammatory markers, and BW management are presented in Supplemental Table 5. In the GRADE Working Group evidence level rates, we found very low quality for QUICKI; low quality for HC, CRP, E-selectin, VCAM-1, and ICAM-1; moderate quality for TG, insulin, HOMA-IR, and WC; and high quality for TC, LDL cholesterol, HDL cholesterol, SBP, DBP, FBG, BW, and BMI.

### Publication bias assessment

**FIGURE 6:**
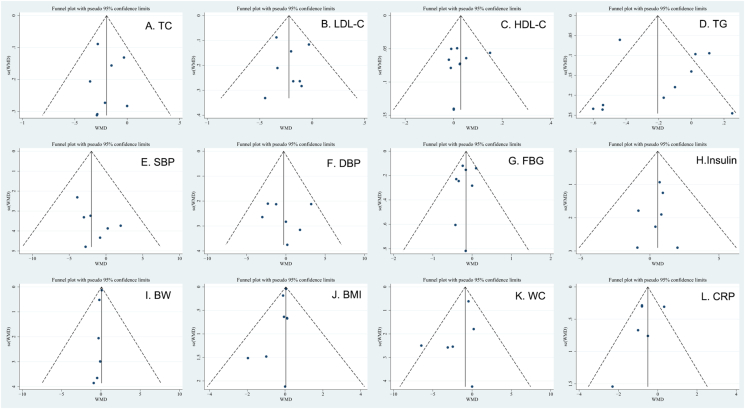
Funnel plot demonstrating publication bias in the studies reporting the impact of hesperidin on CVDRFs: (A) TC; (B) LDL cholesterol; (C) HDL cholesterol; (D) TG; (E) SBP; (F) DBP; (G) FBG; (H) insulin; (I) BW; (J) BMI; (K) WC; (L) CRP. BW, body weight; CRP, C-reactive protein; DBP, diastolic blood pressure; FBG, fasting blood glucose; HDL, high-density lipoprotein cholesterol; LDL, low-density lipoprotein cholesterol; SBP, systolic blood pressure; TC, total cholesterol; TG, triglyceride; WC, waist circumference.

Based on the funnel plots, we did not discover any significant publication bias (Figure 6). Furthermore, consistent results were obtained using the tests of Begg rank correlation and Egger linear regression (*P* > 0.05) (Supplemental Table 6). Because the number of the included trials was small (*n* ≤ 5), we did not evaluate the publication bias or HOMA-IR or QUICKI, HC, E-selectin, VCAM-1, and ICAM-1.

## Discussion

Hesperidin is a commonly found flavanone and is a potential agent for the modulation and therapy of CVD and its associated complications. Many studies have shown that hesperidin and its derivative chemicals help improve CVDRFs, such as hypertension, adiposity, and glucose and lipid metabolism. Meanwhile, inconsistent conclusions were obtained from different clinical trials. In this review, we summarized the available scientific data on the beneficial effects of hesperidin extracts or purified hesperidin ingestion on the biomarkers associated with CVD risk acquired in RCTs. For statistical pooling, we included 12 trials with 589 individuals. We found that hesperidin positively affects TC, LDL cholesterol, FBG, QUICKI, CRP, VCAM-1, and ICAM-1. However, compared with the control group, the HDL cholesterol, TG, SBP, DBP, insulin, HOMA-IR, BW, BMI, WC, HC, and E-selectin were not changed after hesperidin supplementation treatment. According to our subgroup analysis, hesperidin intake dramatically suppressed DBP, SBP, TG, and TC levels in the following subgroups: hesperidin ≥ 500 mg/d, mean age <50 y, mean BMI ≥30, duration ≥6 weeks, and parallel design. In addition, hesperidin supplementation significantly changed TG, FBG, and WC levels in participants with BMI ≥ 30 and trials with a crossover design (*P <* 0.05). Citrus fruits and juices are widely consumed globally and serve as easily accessible dietary sources of hesperidin. Hesperidin is one of the most promising bioflavonoids, which can be efficiently extracted from the waste residues generated during citrus fruit processing, making it economically advantageous [48]. Hesperidin supplements, either alone or in combination with other citrus bioflavonoids, are commercially available. Hence, consuming hesperidin might be a beneficial dietary strategy for reducing hyperglycemia and hypercholesterolemia, especially in particular patient subgroups.

Lipoprotein and lipid metabolism dysregulation is significantly connected to the pathogenesis of many human disorders, such as CVDs [49]. Observational epidemiologic evidence indicates that individuals with hyperlipidemia have a 3-fold higher risk of heart attack than the general population with normal lipid concentrations, and a 1% reduction in serum cholesterol is strongly correlated with a 3% decrease in CVD risk [4,50]. The advantages of lowering LDL cholesterol, with a 1% reduction being linked to a corresponding 1% decrease in CVD events [51]. The main goal of drug therapy is to gradually attain lower LDL cholesterol levels to address cholesterol issues and prevent CVD. In this study, we found that hesperidin modifies dyslipidemia and subsequently affects atherosclerosis, exhibiting lipid-lowering effects. Hesperidin might also decrease cholesterol, although the exact processes underlying these effects are not entirely understood. Hesperidin can favor lipid metabolism by inhibiting the activities of phosphatidate phosphohydrolase, glucose-6-phosphate dehydrogenase, and hepatic fatty acid synthase [52]. Moreover, hesperidin treatment decreases plasma and hepatic cholesterol concentrations by downregulating the activities of HMG-CoA reductase and ACAT [53]. Hesperidin administration also decreases lipid synthesis while increasing lipolysis in adipocytes prepared from human mesenchymal stem cells [54].

Moreover, we showed that participants treated with hesperidin had statistically significantly lower FBG, which seems to contradict the previous study [55]. FBG concentration are considered a key variable in the diagnosis of diabetes and are also adopted by the Food and Drug Administration to evaluate the efficacy of dietary supplements and drugs. Hesperidin exerts its glucose-lowering properties by regulating the enzymes in glucose metabolism, enhancing glucokinase activity, and impairing gluconeogenic glucose-6-phosphatase [56,57]. Furthermore, hesperidin has antidiabetic properties by suppressing the formation of advanced glycation end products and oxidative stress and enhancing glucose uptake [58].

Circulating inflammatory indicators are powerful CVD predictors with important roles in atherosclerosis development [59]. CRP is released by the liver and is the most reliable and substantial predictor of CVD risk [60]. Leukocytes and endothelial cells expressing soluble VCAM-1 and ICAM-1 can stimulate the activation and adhesion at the inflammatory site, promoting atherosclerotic processes at the endothelial surface [61]. Plasma soluble ICAM-1 and sVCAM-1 concentrations have been linked to CVD incidence and can be used to predict the risk of cardiovascular events [62]. Selectins, such as sE-selectin and sP-selectin, can participate in ischemia-reperfusion damage, arterial and venous thrombosis, atherosclerosis, and other CVDs by facilitating signal transduction and leukocyte adhesion at the vascular wall [63]. The meta-analysis by Lorzadeh et al. [64] included 6 RCTs and indicated that hesperidin significantly reduces VCAM-1 but not CRP, E-selectin, and ICAM-1. By contrast, we found significant changes in CRP and ICAM-1 after hesperidin consumption.

It should be noted that bioavailability is a critical factor in evaluating the therapeutic effect of oral medicine. Solubility and permeability through biological membranes play a significant role in influencing the bioavailability [65]. Hesperidin has a chiral carbon at position 2, resulting in 2 diastereoisomers, 2R and 2S, with the 2S-diastereoisomer being the prevalent form in nature [66]. Hesperidin presents a low solubility that may affect its absorption and, therefore, its bioavailability. Several studies have shown differences in transport, metabolism, biological effects, and kinetics between the 2 hesperidin diastereoisomers in plasma and urine [[67], [68], [69]]. Thus, the bioavailability of hesperidin is an important issue that must be considered when examining its efficacy. Future research should focus on the relationships between the bioavailability of hesperidin and delivery systems. In addition, there is a need to standardize a hesperidin intervention protocol, including the consistency of dosage, duration of administration, and pharmaceutical dosage forms.

This study is superior to previous systematic reviews and meta-analyses [24,25] for the following reasons: first, we used stringent inclusion and exclusion criteria and considered only RCTs to guarantee the inclusion of high-quality data during synthesis. Therefore, to our knowledge, this meta-analysis was the most recent, critical, in-depth, comparative investigation, which typically redefined and strengthened earlier study conclusions. Second, we conducted several subgroup analyses based on mean age, study design, baseline BMI, intervention duration, and hesperidin dose to investigate the effects of hesperidin intervention across various factors. Third, we performed the analyses of sensitivity and publication bias to obtain more objective decisions and did not find substantial publication bias. Finally, we used restricted maximum likelihood–based dose–response analysis to find an optimal duration and dosage for hesperidin intervention. In addition, we conducted the criteria of GRADE to evaluate the clinical evidence’s overall body and the recommendation’s strength for each outcome, different from previous studies.

Although this updated meta-analysis has promising results, some limitations are also present. First, the bias of publication was not significant using both Begg rank correlation tests and Egger linear regression. However, we found significant heterogeneity among trials for TG (*I*^2^ = 80%), QUICKI (*I*^2^ = 99%), CRP (*I*^2^ = 56%), E-selectin (*I*^2^ = 59%), VCAM-1 (*I*^2^ = 87%), and ICAM-1 (*I*^2^ = 62%) outcomes. The potential causes of the heterogeneity are likely attributable to variations in study quality, the dosage of hesperidin consumed, dietary habits, and individual characteristics such as age, sex, and baseline BMI. Sensitivity, meta-regression, and a priori subgroup analyses did not completely solve the sources of heterogeneity. Second, the studies included in this meta-analysis, specifically on some CVD markers such as HC, E-selectin, VCAM-1, and ICAM-1, were not enough, providing inconclusive results. Therefore, these findings must be interpreted cautiously, and more large-scale, high-quality clinical studies are required to get conclusive results. Third, the habits of consumption is believed to affect the lipid profile, blood pressure, and other CVD risk factors significantly in clinics. Because the intervention periods of the included studies were very short (3–12 wk), we could not assess the long-term effect of hesperidin therapy on CVDRFs. Finally, despite the included studies being designed as RCTs, most were classified as unclear based on our risk-of-bias assessment, implying that more research might significantly impact the confidence in the estimated effects.

In conclusion, this meta-analysis of RCTs indicated that citrus flavanone hesperidin supplementation could significantly improve TC, LDL cholesterol, FBG, QUICKI, CRP, VCAM-1, and ICAM-1. However, no beneficial effect were found in HDL cholesterol, TG, SBP, DBP, insulin, HOMA-IR, BW, BMI, WC, HC, or E-selectin. The changes in CVD biomarkers were affected by the trial design, supplement dosage, mean age, baseline BMI, and intervention duration. Our findings might help health care practitioners and the general public better comprehend the data on the CVD preventive benefits of hesperidin. Nevertheless, further long-term clinical studies with more homogenous doses and bigger populations are recommended to get a more exact and comprehensive conclusion on the impact of hesperidin on CVDRFs.

## Author contributions

The authors’ responsibilities were as follows – HH: conceived and designed the study, performed acquisition of data, analysis and interpretation of data, drafted the manuscript, discussed the idea of the meta-analysis, submitted the paper; DL, BH: completed the database searches and selected, reviewed the articles, and extracted the data; GZ, YC: reviewed and extracted the data and performed the data analyses; and all authors: read and approved the final version of the manuscript.

## Conflict of interest

The authors declare that they have no competing interest.

## Funding

This work was supported by the Traditional Medicine Research Program of Guangdong Province (Grant No. 20211410) and Medical Scientific Research Foundation of Guangdong Province (Grant No. A2021356).

## Data availability

The data sets used and/or analyzed during this study are available from the corresponding author on reasonable request.

## Appendix A Supplementary data

Multimedia component1 Multimedia component1
