# Supplementary material for: Effects of Citrus Flavanone Hesperidin Extracts or Purified Hesperidin Consumption on Risk Factors for Cardiovascular Disease: Evidence From an Updated Meta-analysis of Randomized Controlled Trials
Source: Curr Dev Nutr. 2023 Dec 9;8(9):102055. doi: 10.1016/j.cdnut.2023.102055 (PMC11399677; doi:10.1016/j.cdnut.2023.102055)
Supplement: Multimedia component1 [file mmc1.docx]

**Effects of citrus flavanone hesperidin extracts or purified hesperidin consumption on risk factors for cardiovascular disease: evidence from an updated meta-analysis of randomized controlled trials**

***Running title:*** Hesperidin consumption on CVDRFs

Haohai Huang^1, 2, *^ , Dan Liao^3^, Bin He^2^, Guanghui Zhou^4^, Yejia Cui^5^

***Authors' Affiliations:***

*^1^**Department of Clinical Pharmacy, Dongguan Songshan Lake Central Hospital, Affiliated Dongguan Shilong People’s Hospital of Southern Medical University, Dongguan, Guangdong, China*

*^2^Medical and Pharmacy Research Laboratory, Dongguan Songshan Lake Central Hospital, Affiliated Dongguan Shilong People’s Hospital of Southern Medical University, Dongguan, Guangdong, China*

*^3^Department of Gynaecology, Dongguan Songshan Lake Central Hospital, Affiliated Dongguan Shilong People’s Hospital of Southern Medical University, Dongguan, Guangdong, China*

*^4^Department of Rehabilitation Medicine, Dongguan Songshan Lake Central Hospital, Affiliated Dongguan Shilong People’s Hospital of Southern Medical University, Dongguan, Guangdong, China*

*^5^Department of Clinical Laboratory, Dongguan Songshan Lake Central Hospital, Affiliated Dongguan Shilong People's Hospital of Southern Medical University, Dongguan, Guangdong, China*

***Address for correspondence:**

Haohai Huang, Department of Clinical Pharmacy, Medical and Pharmacy Research Laboratory, Dongguan Songshan Lake Central Hospital, Affiliated Dongguan Shilong People’s Hospital of Southern Medical University, No.1, Huangzhou Xianglong Road of Shilong Town, Dongguan, Guangdong, 523326, China.

E-mail address: [haohaihuang@hotmail.com](mailto:haohaihuang@hotmail.com) (H. Huang)

**Supplementary Material**

**Supplemental Table S1** Literature search strategy

Cochrane Central Register of Controlled Trials (CENTRAL), MEDLINE/PubMed, Google Scholar, Scopus, Web of Science, and EMBASE database will be searched for studies from database inception to March 2023 using the following complimentary strategies without language restrictions.

The following Medical Subjects Headings (MeSH) and free-text terms were used:

#1 AND (#2 OR #3 OR #4 OR #5 OR #6 OR #7) AND #8

| #1 Hesperidin related terms:  “hesperidin” OR “hesperetin” OR “citrus flavanone” |
| --- |
| #2 Cardiovascular disease related terms:  “cardiovascular” OR “coronary heart disease” OR “cardiovascular event” OR “ischemic heart disease” |
| #3 Weight related terms:  "body mass index" OR “BMI” OR "Overweight" OR “obesity” OR "central obesity" OR "abdominal obesity" OR adiposity OR "waist circumference" OR "hip circumference" OR "waist-hip ratio" OR Obese OR "weight change" OR weight OR "body weight" OR "weight loss" OR "abdominal fat" OR "body fat" OR "fat mass" OR “fat free mass” OR “mid-upper arm circumference” OR “adipose tissue” OR “Quetelet index” |
| #4 Blood pressure related terms:  “blood pressure” OR “BP” OR “diastolic pressure” OR “systolic pressure” OR “pulse pressure” OR “hypertension” OR “arterial pressure” OR “arterial tension” OR “systolic blood pressure” OR “diastolic blood pressure” OR “SBP” OR “DBP” OR “MAP” OR “arterial blood pressure” OR “aortic plus pressure” OR “aortic pressure” OR “aortic tension” OR “systolic arterial pressure” |
| #5 Lipid profile related terms:  “cholesterol” OR “triglyceride” OR “LDL” OR “LDL-C” OR “LDL-cholesterol” OR “HDL” OR “HDL-C” OR“HDL-cholesterol” OR “hyperlipidemia” OR “hyperlip-idemic” OR “hypolipidemic” OR “dyslipidemia” OR “dyslipidemic” |
| #6 Glycemic indices related terms:  “glycemic” OR “glycemic indices” OR “glucose” OR “blood glucose” OR “blood sugar” OR “fast plasma glucose” OR “FPG” OR “fasting blood sugar” OR “FBS” OR “insulin” OR “hyperinsulinism” OR “hyperinsulinemia” OR “insulin resistance” OR “insulin sensitivity”, “hemoglobin A" OR “glycosylated” OR “glycosylated hemoglobin” OR “glycated hemoglobins” OR “glycosylated hemoglobin A" OR “glycohemoglobin A" OR “HbA1” OR “hyperglycemia” OR “hyperglycemias” OR “hypoglycemia” OR “hypoglycemias” OR “fasting hypoglycemia" OR “glycemic load” |
| #7 Inflammation related terms:  “cytokine” OR “tumor necrosis factor” OR “interleukin” OR “C-reactive protein” OR “fibrinogen” OR “adiponectin” OR “selectins” OR “adhesion molecule” OR “TNF” OR “IL” OR “CRP” OR “ICAM-1” OR “VCAM-1” OR “PAI-1” |
| #8 Types of article related terms:  “randomized controlled trial" OR “randomized" OR “randomly" OR "trials" OR "trial" OR "clinical trials" OR "placebo" AND “RCT” OR “crossover” OR “parallel” |
| Filters:  Humans |

**Supplemental Table S2** Subgroup analyses of the effects of Hesperidin consumption on CVD risk factors.

| Variables | Study design | |  | Mean age | |  | Baseline BMI | |  | Hesperidin Dose | |  | Intervention Duration | |
| --- | --- | --- | --- | --- | --- | --- | --- | --- | --- | --- | --- | --- | --- | --- |
|  | Parallel | Crossover |  | ＜50 years old | ≥50 years old |  | ＜30 kg/m^2^ | ≥30 kg/m^2^ |  | ＜500 mg/d | ≥500 mg/d |  | ＜6 weeks | ≥6 weeks |
| ***TC (mmol/L)*** |  |  |  |  |  |  |  |  |  |  |  |  |  |  |
| No. of trials | 6 | 3 |  | 4 | 5 |  | 5 | 4 |  | 2 | 7 |  | 4 | 5 |
| WMD (95% CI) | -0.20  [-0.32, -0.08] | -0.16  [-0.48, 0.16] |  | -0.26  [-0.39, -0.12] | -0.07  [-0.27, 0.12] |  | -0.08  [-0.25, 0.09] | -0.28  [-0.43, -0.14] |  | 0.00  [-0.39, 0.39] | -0.21  [-0.33, -0.10] |  | -0.08  [-0.29, 0.12] | -0.24  [-0.38, -0.11] |
| *P* | 0.001* | 0.33 |  | 0.0002* | 0.45 |  | 0.35 | 0.0002* |  | 1.00 | 0.0003* |  | 0.42 | 0.0004* |
| I^2^ (%) | 0 | 0 |  | 0 | 0 |  | 0 | 0 |  | 0 | 0 |  | 0 | 0 |
| ***LDL-C (mmol/L)*** |  |  |  |  |  |  |  |  |  |  |  |  |  |  |
| No. of trials | 6 | 3 |  | 4 | 5 |  | 5 | 4 |  | 2 | 7 |  | 4 | 5 |
| WMD (95% CI) | -0.23  [-0.35, -0.11] | -0.13  [-0.44, 0.17] |  | -0.29  [-0.43, -0.16] | -0.09  [-0.27, 0.08] |  | -0.12  [-0.27, 0.04] | -0.31  [-0.45, -0.16] |  | -0.10  [-0.49, 0.29] | -0.23  [-0.34, -0.12] |  | -0.09  [-0.28, 0.09] | -0.28  [-0.41, -0.15] |
| *P* | 0.0003* | 0.39 |  | 0.0001* | 0.30 |  | 0.14 | 0.0001* |  | 0.62 | 0.0001* |  | 0.33 | 0.0001* |
| I^2^ (%) | 8 | 0 |  | 0 | 0 |  | 0 | 0 |  | 0 | 0 |  | 0 | 0 |
| ***HDL-C (mmol/L)*** |  |  |  |  |  |  |  |  |  |  |  |  |  |  |
| No. of trials | 6 | 4 |  | 5 | 5 |  | 5 | 5 |  | 2 | 8 |  | 4 | 6 |
| WMD (95% CI) | 0.04  [-0.02, 0.09] | 0.01  [-0.07, 0.09] |  | 0.01  [-0.04, 0.06] | 0.05  [-0.02, 0.13] |  | 0.04  [-0.04, 0.11] | 0.02  [-0.03, 0.08] |  | 0.00  [-0.20, 0.20] | 0.03  [-0.01, 0.07] |  | 0.05  [-0.04, 0.14] | 0.01  [-0.04, 0.06] |
| *P* | 0.20 | 0.75 |  | 0.64 | 0.16 |  | 0.38 | 0.45 |  | 1.00 | 0.16 |  | 0.23 | 0.65 |
| I^2^ (%) | 17 | 0 |  | 0 | 13 |  | 31 | 0 |  | 0 | 0 |  | 32 | 0 |
| ***TG (mmol/L)*** |  |  |  |  |  |  |  |  |  |  |  |  |  |  |
| No. of trials | 6 | 4 |  | 5 | 5 |  | 5 | 5 |  | 2 | 8 |  | 4 | 6 |
| WMD (95% CI) | -0.11  [-0.37, 0.16] | -0.31  [-0.54, -0.08] |  | -0.39  [-0.63, -0.16] | 0.02  [-0.08, 0.13] |  | 0.05  [-0.06, 0.16] | -0.44  [-0.54, -0.33] |  | -0.04  [-0.25, 0.18] | -0.22  [-0.45, 0.01] |  | 0.03  [-0.09, 0.15] | -0.31  [-0.56, -0.07] |
| *P* | 0.42 | 0.009* |  | 0.001* | 0.66 |  | 0.37 | 0.0001* |  | 0.73 | 0.06 |  | 0.63 | 0.01* |
| I^2^ (%) | 87 | 22 |  | 54 | 0 |  | 0 | 0 |  | 0 | 83 |  | 0 | 70 |
| ***SBP (mmHg)*** |  |  |  |  |  |  |  |  |  |  |  |  |  |  |
| No. of trials | 3 | 4 |  | 2 | 5 |  | 4 | 3 |  | 2 | 5 |  | 3 | 4 |
| WMD (95% CI) | -2.86  [-6.20, 0.47] | -0.83  [-4.65, 2.98] |  | -2.31  [-7.57, 2.95] | -1.89  [-4.74, 0.97] |  | -2.59  [-5.69, 0.52] | -0.84  [-5.11, 3.43] |  | -2.19  [-7.35, 2.97] | -1.92  [-4.79, 0.96] |  | 0.61  [-3.87, 5.09] | -3.17  [-6.21, -0.14] |
| *P* | 0.09 | 0.67 |  | 0.39 | 0.20 |  | 0.10 | 0.70 |  | 0.41 | 0.19 |  | 0.79 | 0.04 |
| I^2^ (%) | 0 | 0 |  | 0 | 0 |  | 0 | 0 |  | 0 | 0 |  | 0 | 0 |
| ***DBP (mmHg)*** |  |  |  |  |  |  |  |  |  |  |  |  |  |  |
| No. of trials | 3 | 4 |  | 2 | 5 |  | 4 | 3 |  | 2 | 5 |  | 3 | 4 |
| WMD (95% CI) | -0.55  [-4.50, 3.40] | -0.16  [-2.88, 2.57] |  | -0.90  [-4.52, 2.73] | -0.07  [-2.55, 2.40] |  | -0.10  [-3.23, 3.04] | -0.63  [-3.66, 2.41] |  | -0.91  [-5.57, 3.76] | -0.08  [-2.18, 2.03] |  | 2.00  [-0.93, 4.93] | -1.83  [-4.24, 0.58] |
| *P* | 0.78 | 0.91 |  | 0.63 | 0.95 |  | 0.95 | 0.69 |  | 0.70 | 0.94 |  | 0.18 | 0.14 |
| I^2^ (%) | 57 | 0 |  | 0 | 22 |  | 41 | 0 |  | 26 | 0 |  | 0 | 0 |
| ***FBG (mmol/L)*** |  |  |  |  |  |  |  |  |  |  |  |  |  |  |
| No. of trials | 4 | 4 |  | 4 | 4 |  | 3 | 5 |  | 2 | 5 |  | 2 | 5 |
| WMD (95% CI) | -0.12  [-0.29, 0.06] | -0.27  [-0.55, -0.01] |  | -0.25  [-0.41, -0.09] | 0.05  [-0.18, 0.29] |  | 0.06  [-0.18, 0.30] | -0.25  [-0.41, -0.09] |  | 0.08  [-0.17, 0.33] | -0.25  [-0.41, -0.09] |  | -0.02  [-0.54, 0.51] | -0.17  [-0.32, -0.01] |
| *P* | 0.21 | 0.04* |  | 0.002* | 0.65 |  | 0.63 | 0.002* |  | 0.52 | 0.002* |  | 0.95 | 0.03* |
| I^2^ (%) | 20 | 0 |  | 0 | 0 |  | 0 | 0 |  | 0 | 0 |  | 0 | 13 |
| ***Insulin (µU/mL)*** |  |  |  |  |  |  |  |  |  |  |  |  |  |  |
| No. of trials | 3 | 4 |  | 4 | 3 |  | 2 | 5 |  | 2 | 5 |  | 2 | 5 |
| WMD (95% CI) | 0.87  [-0.55, 2.29] | -0.07  [-2.13, 2.00] |  | 0.58  [-0.69, 1.85] | 0.49  [-2.49, 3.47] |  | 1.01  [-2.49, 4.51] | 0.51  [-0.73, 1.75] |  | 1.01  [-2.49, 4.51] | 0.51  [-0.73, 1.75] |  | -0.09  [-3.60, 3.41] | 0.65  [-0.59, 1.89] |
| *P* | 0.23 | 0.95 |  | 0.37 | 0.75 |  | 0.57 | 0.42 |  | 0.57 | 0.42 |  | 0.96 | 0.30 |
| I^2^ (%) | 0 | 0 |  | 0 | 0 |  | 0 | 0 |  | 0 | 0 |  | 0 | 0 |
| ***HOMA-IR*** |  |  |  |  |  |  |  |  |  |  |  |  |  |  |
| No. of trials | 3 | 2 |  | 4 | 1 |  | 4 | 1 |  | 0 | 5 |  | 0 | 5 |
| WMD (95% CI) | 0.08  [-0.37, 0.52] | -0.07  [-0.78, 0.64] |  | 0.03  [-0.37, 0.43] | 0.05  [-1.14, 1.24] |  | 0.03  [-0.37, 0.43] | 0.05  [-1.14, 1.24] |  | / | 0.03  [-0.34, 0.41] |  | / | 0.03  [-0.34, 0.41] |
| *P* | 0.74 | 0.85 |  | 0.87 | 0.93 |  | 0.87 | 0.93 |  | / | 0.86 |  | / | 0.86 |
| I^2^ (%) | 0 | 0 |  | 0 | NA |  | 0 | NA |  | / | 0 |  | / | 0 |
| ***QUICKI*** |  |  |  |  |  |  |  |  |  |  |  |  |  |  |
| No. of trials | 2 | 3 |  | 3 | 2 |  | 1 | 4 |  | 1 | 4 |  | 1 | 4 |
| WMD (95% CI) | 0.13  [-0.13, 0.40] | 0.01  [0.00, 0.01] |  | 0.00  [-0.00, 0.01] | 0.14  [-0.13, 0.40] |  | 0.27  [0.24, 0.30] | 0.00  [-0.00, 0.01] |  | 0.27  [0.24, 0.30] | 0.00  [-0.00, 0.01] |  | 0.00  [-0.01, 0.01] | 0.07  [0.01, 0.13] |
| *P* | 0.32 | 0.04* |  | 0.18 | 0.31 |  | 0.0001* | 0.14 |  | 0.0001* | 0.14 |  | 0.58 | 0.03* |
| I^2^ (%) | 100 | 0 |  | 38 | 100 |  | NA | 8 |  | NA | 8 |  | NA | 99 |
| ***BW (kg)*** |  |  |  |  |  |  |  |  |  |  |  |  |  |  |
| No. of trials | 6 | 1 |  | 3 | 4 |  | 5 | 2 |  | 0 | 7 |  | 2 | 5 |
| WMD (95% CI) | 0.08  [-0.18, 0.33] | -0.92  [-8.48, 6.64] |  | -0.22  [-1.22, 0.79] | 0.10  [-0.17, 0.36] |  | 0.08  [-0.18, 0.33] | -0.69  [-5.89, 4.51] |  | / | 0.08  [-0.18, 0.33] |  | 0.10  [-0.17, 0.36] | -0.21  [-1.19, 0.77] |
| *P* | 0.55 | 0.84 |  | 0.67 | 0.47 |  | 0.54 | 0.80 |  | / | 0.55 |  | 0.47 | 0.67 |
| I^2^ (%) | 0 | NA |  | 0 | 0 |  | 0 | 0 |  | / | 0 |  | 0 | 0 |
| ***BMI (kg/m^2^)*** |  |  |  |  |  |  |  |  |  |  |  |  |  |  |
| No. of trials | 6 | 2 |  | 3 | 5 |  | 5 | 3 |  | 0 | 8 |  | 3 | 5 |
| WMD (95% CI) | -0.02  [-0.56, 0.52] | -0.67  [-3.05, 1.71] |  | -1.14  [-2.94, 0.66] | 0.05  [-0.50, 0.60] |  | 0.04  [-0.50, 0.59] | -1.18  [-3.04, 0.68] |  | / | -0.05  [-0.58, 0.47] |  | 0.02  [-0.66, 0.70] | -0.15  [-0.98, 0.67] |
| *P* | 0.94 | 0.58 |  | 0.22 | 0.87 |  | 0.87 | 0.21 |  | / | 0.84 |  | 0.96 | 0.71 |
| I^2^ (%) | 0 | 0 |  | 0 | 0 |  | 0 | 0 |  | / | 0 |  | 0 | 0 |
| ***WC (cm)*** |  |  |  |  |  |  |  |  |  |  |  |  |  |  |
| No. of trials | 3 | 3 |  | 4 | 2 |  | 2 | 4 |  | 0 | 6 |  | 2 | 4 |
| WMD (95% CI) | -0.52  [-1.64, 0.59] | -3.77  [-7.19, -0.34] |  | -2.47  [-5.25, 0.30] | 0.17  [-3.07, 3.41] |  | -0.39  [-1.53, 0.75] | -3.61  [-6.33, -0.90] |  | / | -1.45  [-3.23, 0.33] |  | 0.17  [-3.07, 3.41] | -2.47  [-5.25, 0.30] |
| *P* | 0.36 | 0.03* |  | 0.08 | 0.92 |  | 0.50 | 0.009* |  | / | 0.11 |  | 0.92 | 0.08 |
| I^2^ (%) | 0 | 0 |  | 54 | 0 |  | 0 | 0 |  | / | 28 |  | 0 | 54 |
| ***CRP (mg/L)*** |  |  |  |  |  |  |  |  |  |  |  |  |  |  |
| No. of trials | 4 | 2 |  | 3 | 3 |  | 3 | 3 |  | 0 | 6 |  | 2 | 4 |
| WMD (95% CI) | -0.51  [-1.14, 0.12] | -0.90  [-2.36, 0.57] |  | -0.80  [-1.28, -0.31] | -0.45  [-1.52, 0.63] |  | -0.80  [-1.28, -0.31] | -0.45  [-1.52, 0.63] |  | / | -0.56  [-1.11, -0.01] |  | -0.55  [-2.98, 1.88] | -0.80  [-1.17, -0.42] |
| *P* | 0.11 | 0.23 |  | 0.001* | 0.42 |  | 0.001* | 0.42 |  | / | 0.04* |  | 0.66 | 0.0001* |
| I^2^ (%) | 70 | 9 |  | 0 | 77 |  | 0 | 77 |  | / | 56 |  | 64 | 0 |

WMD, weighted mean differences; CI, confidence interval; NA, not available; TC, total cholesterol; LDL-C, low-density lipoprotein cholesterol; HDL-C, high-density lipoprotein cholesterol; TG, triglycerides; SBP, systolic blood pressure; DBP, diastolic blood pressure; FBG, fasting blood glucose; HOMA-IR, homeostatic model assessment of insulin resistance; QUICKI, Quantitative insulin-sensitivity check index; BW, body weight; BMI, Body Mass Index; WC, waist circumference; CRP, C-reactive protein; *Indicates a significant result

**Supplemental Table S3** Sensitivity analyses of hesperidin consumption on CVDRFs by using a fixed-effects model

| Outcomes | No. of trials | No. of patients | WMD (95% CI) | *P* Value |
| --- | --- | --- | --- | --- |
| TC | 9 | 531 | -0.20 [-0.31, -0.08] | 0.0006* |
| LDL-C | 9 | 531 | -0.22 [-0.33, -0.11] | 0.0001* |
| HDL-C | 10 | 575 | 0.03 [-0.01, 0.07] | 0.17 |
| TG | 10 | 575 | -0.18 [-0.38, 0.00] | 0.05 |
| SBP | 7 | 389 | -1.98 [-4.49, 0.53] | 0.12 |
| DBP | 7 | 389 | -0.28 [-2.14, 1.58] | 0.77 |
| FBG | 8 | 407 | -0.15 [-0.29, -0.02] | 0.02* |
| Insulin | 7 | 347 | 0.57 [-0.60, 1.74] | 0.34 |
| HOMA-IR | 5 | 246 | 0.03 [-0.34, 0.41] | 0.86 |
| QUICKI | 5 | 249 | 0.01 [0.01, 0.01] | 0.001* |
| BW | 7 | 442 | 0.08 [-0.18, 0.33] | 0.55 |
| BMI | 8 | 484 | -0.05 [-0.58, 0.47] | 0.84 |
| WC | 6 | 289 | -0.87 [-1.93, 0.18] | 0.10 |
| HC | 2 | 105 | -0.73 [-1.69, 0.22] | 0.13 |
| CRP | 6 | 324 | -0.50 [-0.82, -0.18] | 0.002* |
| VCAM-1 | 3 | 144 | -16.05 [-20.59, -11.52] | 0.001* |
| ICAM-1 | 2 | 96 | -10.50 [-13.26, -7.73] | 0.001* |
| E-selectin | 3 | 171 | -2.80 [-6.14, 0.56] | 0.06 |

*Indicates a significant result

TC, total cholesterol; LDL-C, low-density lipoprotein cholesterol; HDL-C, high-density lipoprotein cholesterol; TG, triglycerides; SBP, systolic blood pressure; DBP, diastolic blood pressure; FBG, fasting blood glucose; HOMA-IR, homeostatic model assessment of insulin resistance; QUICKI, Quantitative insulin-sensitivity check index; BW, body weight; BMI, Body Mass Index; WC, waist circumference; HC, hip circumference; CRP, C-reactive protein; ICAM-1, intercellular adhesion molecule 1; VCAM-1, vascular cell adhesion molecule 1.

**Supplemental Table S4** Meta-regression analysis of the association between changes in CVDRFs with dose and duration of hesperidin supplementation.

| Outcomes | Parameter | β-coefficient | 95% CI | *P* |
| --- | --- | --- | --- | --- |
| TC | Dose | -0.0003 | -0.0009 to 0.0003 | 0.246 |
|  | Duration | -0.0221 | -0.0581 to 0.0139 | 0.190 |
| LDL-C | Dose | -0.0002 | -0.0008 to 0.0004 | 0.396 |
|  | Duration | -0.0257 | -0.0583 to 0.0089 | 0.126 |
| HDL-C | Dose | -.00002 | -0.0002 to 0.0002 | 0.797 |
|  | Duration | -0.0055 | -0.0178 to 0.0068 | 0.335 |
| TG | Dose | -0.0007 | -0.0014 to -0.0008 | 0.031* |
|  | Duration | -0.0563 | -0.0841 to -0.0286 | 0.002* |
| SBP | Dose | -0.0005 | -0.1489 to 0.0138 | 0.931 |
|  | Duration | -0.2634 | -1.3221 to 0.7952 | 0.551 |
| DBP | Dose | -0.0005 | -0.0121 to 0.1095 | 0.903 |
|  | Duration | -0.2484 | -1.0148 to 0.5179 | 0.443 |
| FBG | Dose | -0.0005 | -0.0011 to 0.0001 | 0.087 |
|  | Duration | -0.0447 | -0.0998 to 0.0103 | 0.094 |
| Insulin | Dose | 0.0008 | -0.0067 to 0.0085 | 0.785 |
|  | Duration | 0.0848 | -0.4970 to 0.6666 | 0.723 |
| BW | Dose | 0.0009 | -0.0034 to 0.0053 | 0.616 |
|  | Duration | -0.0397 | -0.2099 to 0.1305 | 0.575 |
| BMI | Dose | 0.0003 | -0.0010 to 0.0017 | 0.582 |
|  | Duration | -0.0245 | -0.0815 to 0.0324 | 0.332 |
| WC | Dose | -0.0070 | -0.0158 to 0.0017 | 0.089 |
|  | Duration | -0.2998 | -1.1615 to 0.5618 | 0.389 |
| CRP | Dose | -0.0005 | -0.0042 to 0.0030 | 0.688 |
|  | Duration | -0.0754 | -0.2699 to 0.1190 | 0.342 |

*Indicates a significant result

***Abbreviations:*** TC, total cholesterol; LDL-C, low-density lipoprotein cholesterol; HDL-C, high-density lipoprotein cholesterol; TG, triglycerides; SBP, systolic blood pressure; DBP, diastolic blood pressure; FBG, fasting blood glucose; BW, body weight; BMI, Body Mass Index; WC, waist circumference; CRP, C-reactive protein.

**Supplemental Table S5** GRADE Evidence Profile.

| **Outcomes** | **No of Participants (studies)** Follow up | **Quality of the evidence** (GRADE) | **Anticipated absolute effects** | |
| --- | --- | --- | --- | --- |
|  |  |  |  | |
|  |  |  | **Risk with Control** | **Risk difference with Hesperidin** (95% CI) |
| **TC** | 531 (9 studies) 3 to 12 weeks | ⊕⊕⊕⊕ **HIGH** |  | The mean tc in the intervention groups was **0.2 lower** (0.31 to 0.08 lower) |
| **LDL-C** | 531 (9 studies) 3 to 12 weeks | ⊕⊕⊕⊕ **HIGH** |  | The mean ldl-c in the intervention groups was **0.22 lower** (0.33 to 0.11 lower) |
| **HDL-C** | 575 (10 studies) 3 to 12 weeks | ⊕⊕⊕⊕ **HIGH** |  | The mean hdl-c in the intervention groups was **0.03 higher** (0.01 lower to 0.07 higher) |
| **TG** | 575 (10 studies) 3 to 12 weeks | ⊕⊕⊕⊝ **MODERATE** |  | The mean tg in the intervention groups was **0.18 lower** (0.38 lower to 0.01 higher) |
| **SBP** | 389 (7 studies) 3 to 12 weeks | ⊕⊕⊕⊕ **HIGH** |  | The mean sbp in the intervention groups was **1.98 lower** (4.49 lower to 0.53 higher) |
| **DBP** | 389 (7 studies) 3 to 12 weeks | ⊕⊕⊕⊕ **HIGH** |  | The mean dbp in the intervention groups was **0.28 lower** (2.14 lower to 1.58 higher) |
| **FBG** | 407 (8 studies) 3-12 weeks | ⊕⊕⊕⊕ **HIGH** |  | The mean fbg in the intervention groups was **0.15 lower** (0.29 to 0.02 lower) |
| **Insulin** | 347 (7 studies) 6-12 weeks | ⊕⊕⊕⊝ **MODERATE** |  | The mean insulin in the intervention groups was **0.5 higher** (0.69 lower to 1.7 higher) |
| **HOMA-IR** | 246 (5 studies) 3 to 12 weeks | ⊕⊕⊕⊝ **MODERATE** |  | The mean homa-ir in the intervention groups was **0.03 higher** (0.34 lower to 0.41 higher) |
| **QUICKI** | 249 (5 studies) 3 to 12 weeks | ⊕⊝⊝⊝ **VERY LOW** |  | The mean quicki in the intervention groups was **0.06 higher** (0.01 to 0.1 higher) |
| **BW** | 442 (7 studies) 4 to 12 weeks | ⊕⊕⊕⊕ **HIGH** |  | The mean body weight in the intervention groups was **0.08 higher** (0.18 lower to 0.33 higher) |
| **BMI** | 484 (8 studies) 3 to 12 weeks | ⊕⊕⊕⊕ **HIGH** |  | The mean bmi in the intervention groups was **0.05 lower** (0.58 lower to 0.47 higher) |
| **WC** | 289 (6 studies) 2 to 12 weeks | ⊕⊕⊕⊝ **MODERATE** |  | The mean wc in the intervention groups was **1.45 lower** (3.23 lower to 0.33 higher) |
| **HC** | 105 (2 studies) 4 to 12 weeks | ⊕⊕⊝⊝ **LOW** |  | The mean hc in the intervention groups was **0.73 lower** (1.69 lower to 0.22 higher) |
| **CRP** | 324 (6 studies) 3 to 12 weeks | ⊕⊕⊝⊝ **LOW** |  | The mean crp in the intervention groups was **0.56 lower** (1.11 to 0.01 lower) |
| **E-selectin** | 171 (3 studies) 3 to 6 weeks | ⊕⊕⊝⊝ **LOW** |  | The mean e-selectin in the intervention groups was **2.25 lower** (6.14 lower to 1.64 higher) |
| **VCAM-1** | 144 (3 studies) 3 to 6 weeks | ⊕⊕⊝⊝ **LOW** |  | The mean vcam-1 in the intervention groups was **15.6 lower** (30.13 to 1.06 lower) |
| **ICAM-1** | 96 (2 studies) 4 to 6 weeks | ⊕⊕⊝⊝ **LOW** |  | The mean icam-1 in the intervention groups was **13.6 lower** (23.72 to 3.48 lower) |

*The basis for the assumed risk (e.g. the median control group risk across studies) is provided in footnotes. The corresponding risk (and its 95% confidence interval) is based on the assumed risk in the comparison group and the relative effect of the intervention (and its 95% CI).

***Abbreviations:***

CI, Confidence interval; TC, total cholesterol; LDL-C, low-density lipoprotein cholesterol; HDL-C, high-density lipoprotein cholesterol; TG, triglycerides; SBP, systolic blood pressure; DBP, diastolic blood pressure; FBG, fasting blood glucose; HOMA-IR, homeostatic model assessment of insulin resistance; QUICKI, Quantitative insulin-sensitivity check index; BW, body weight; BMI, Body Mass Index; WC, waist circumference; HC, hip circumference; CRP, C-reactive protein; ICAM-1, intercellular adhesion molecule 1; VCAM-1, vascular cell adhesion molecule 1.

GRADE Working Group grades of evidence

**High quality:** Further research is very unlikely to change our confidence in the estimate of effect.

**Moderate quality:** Further research is likely to have an important impact on our confidence in the estimate of effect and may change the estimate.

**Low quality:** Further research is very likely to have an important impact on our confidence in the estimate of effect and is likely to change the estimate.

**Very low quality:** We are very uncertain about the estimate.

**Supplemental Table S6** Publication bias in the meta-analysis of studies

| Outcomes | Begg’s rank correlation test | |  | Egger’s linear regression test | | | |
| --- | --- | --- | --- | --- | --- | --- | --- |
|  | *Z* value | *P* value |  | Intercept (95% CI) | *t* | degrees of freedom | *P* value |
| TC | 0.43 | 0.669 |  | 0.01 (-0.16 to 0.18) | 0.15 | 12 | 0.885 |
| TC | 0.10 | 0.917 |  | 0.34 (-1.03 to 1.71) | 0.59 | 8 | 0.575 |
| LDL-C | 0.31 | 0.754 |  | 0.33 (-1.26 to 1.93) | 0.50 | 8 | 0.635 |
| HDL-C | 0 | 1.000 |  | 0.05 (-0.09 to 0.20) | 0.88 | 9 | 0.88 |
| TG | 0.89 | 0.371 |  | 0.63 (-2.96 to 4.23) | 0.41 | 9 | 0.695 |
| SBP | 0.60 | 0.548 |  | 1.55 (-0.73 to 3.82) | 1.75 | 6 | 0.141 |
| DBP | 0.60 | 0.548 |  | 0.37 (-4.75 to 5.49) | 0.19 | 6 | 0.860 |
| FBG | 0.37 | 0.711 |  | -0.41 (-2.25 to 1.42) | -0.55 | 7 | 0.599 |
| Insulin | 0.90 | 0.368 |  | 0.91 (-0.41 to 2.22) | 1.77 | 6 | 0.137 |
| HOMA-IR | 0.24 | 0.806 |  | 0.14 (-0.66 to 0.93) | 0.54 | 4 | 0.624 |
| QUICKI | 2.20 | 0.027 |  | -0.07 (-0.18 to 0.04) | 2.52 | 4 | 0.137 |
| BW | 0.30 | 0.764 |  | -0.21 (-0.45 to 0.02) | 3.71 | 6 | 0.066 |
| BMI | 0.62 | 0.536 |  | -0.43 (-1.00 to 0.13) | 2.46 | 7 | 0.107 |
| WC | 0.75 | 0.452 |  | 0.15 (-2.49 to 2.80) | 0.16 | 5 | 0.880 |
| CRP | 0.38 | 0.707 |  | -0.14 (-1.72 to 1.44) | -0.25 | 5 | 0.816 |

***Abbreviations:*** TC, total cholesterol; LDL-C, low-density lipoprotein cholesterol; HDL-C, high-density lipoprotein cholesterol; TG, triglycerides; SBP, systolic blood pressure; DBP, diastolic blood pressure; FBG, fasting blood glucose; HOMA-IR, homeostatic model assessment of insulin resistance; QUICKI, Quantitative insulin-sensitivity check index; BW, body weight; BMI, Body Mass Index; WC, waist circumference; CRP, C-reactive protein;
